# Feline Calicivirus Infection Manipulates Central Carbon Metabolism

**DOI:** 10.3390/vetsci12020138

**Published:** 2025-02-07

**Authors:** Guangrong Zhao, Hongwei Zhu, Xiu Xue, Chenpei Zhao, Xin Yu, Linlin Jiang, Jingxian Cong, Yang Liu, Yuanlong He, Jianlong Zhang, Xingxiao Zhang

**Affiliations:** 1 School of Life Sciences, Ludong University, Yantai 264025, China; 2 Collaborative Innovation Center for the Pet Infectious Diseases and Public Health in the Middle and Lower Stream Regions of the Yellow River, Yantai 264025, China; 3 School of Basic Medical Sciences, Heze Medical College, Heze 274000, China; 4 Shandong Engineering Research Center for Aquaculture Environment Control, Yantai 264025, China; 5 Shandong Huanong Biopharmaceutical Co., Ltd., Weifang 262600, China

**Keywords:** glycolysis, pentose phosphate pathway, glutamine metabolism, metabolomics

## Abstract

Viruses can manipulate the host metabolism to achieve optimal replication conditions, and central carbon metabolism (CCM) pathways are often crucial in determining viral infections. Feline calicivirus (FCV), a diminutive RNA viral agent, induces upper respiratory tract infections in feline hosts, with highly pathogenic strains capable of precipitating systemic infections and subsequent host cell necrosis, thereby presenting a formidable challenge to feline survival and protection. However, the relationship between FCV and host cell central carbon metabolism (CCM) remains unclear, and the precise pathogenic mechanisms of FCV are yet to be elucidated. Upon FCV infection of Crandell-Rees Feline Kidney (CRFK) cells, an enhanced cellular uptake of glucose and glutamine was observed. Metabolomics analyses disclosed pronounced alterations in the central carbon metabolism of the infected cells. FCV infection was found to augment glycolytic activity while sustaining the tricarboxylic acid (TCA) cycle flux, with cellular ATP levels remaining invariant. Concurrently, both glutamine metabolism and the flux of the pentose phosphate pathway (PPP) were noted to be intensified. The application of various inhibitory agents targeting glycolysis, glutamine metabolism, and the PPP resulted in a significant suppression of FCV proliferation. Experiments involving glucose and glutamine deprivation demonstrated that the absence of either nutrient markedly curtailed FCV replication. Collectively, these findings suggest a critical interplay between central carbon metabolism and FCV proliferation. FCV infection stimulates CRFK cells to augment glucose and glutamine uptake, thereby supplying the necessary metabolic substrates and energy for viral replication. During the infection, glutamine emerges as the primary energy substrate, ensuring ATP production and energy homeostasis, while glucose is predominantly channeled into the pentose phosphate pathway to facilitate nucleotide synthesis.

## Simple Summary

Viruses, functioning as cellular parasites, exhibit a life cycle and metabolic processes that are intricately linked to those of their host organisms. Current investigations have revealed that viruses subvert the metabolic activities of host cells to facilitate their replication. In this study, we employed metabolomic and transcriptomic sequencing techniques to establish that FCV023 infection in CRFK cells elicits a pronounced shift in the host’s central carbon metabolism, with FCV023 demonstrating a distinctive pattern of carbon utilization. The replication of FCV023 was significantly impeded by the application of metabolic inhibitors targeting glycolysis, the pentose phosphate pathway, and glutamine metabolism. Comparative supplementation assays with glutamine and glucose revealed that both are essential carbon sources for optimal viral replication. However, glutamine appears to function as a novel energy substrate within FCV-infected cells, whereas glucose is predominantly channeled into the pentose phosphate pathway to synthesize nucleotides critical for the replication of the viral genome.

## 1. Introduction

Feline calicivirus (FCV) is a highly contagious pathogen and a single-stranded positive-sense RNA virus belonging to the Vesivirus genus of the Caliciviridae family [1,2]. FCV exhibits a broad host range within the Felidae family, infecting all recognized members such as leopards, lions, and tigers. Additionally, there have been documented instances of FCV isolation in canines. This potential for cross-species infectivity and transmissibility presents substantial threats to the viability of both wild and domestic felids. Infection with FCV can induce a spectrum of clinical manifestations in the host, including pyrexia, ulceration, tracheitis, and other upper respiratory tract symptoms. Notably, the virulent systemic variant of feline calicivirus (VS-FCV) is capable of inducing systemic infections and may ultimately result in host lethality [3,4]. FCV is currently endemic worldwide, yet the pathogenic mechanisms of FCV remain unclear. The protective efficacy of existing vaccines against FCV is limited, and there are no specific antiviral drugs available for clinical treatment. Therefore, elucidating the pathogenic mechanisms of FCV is urgent for the development of targeted drugs and the design of novel vaccines, which is of great significance for the prevention, control, and treatment of FCV [5,6].

CCM functions as a pivotal metabolic pathway in organisms, facilitating the acquisition of energy. It enables the swift provision of energy and serves as a source of metabolic precursors essential for the biosynthesis of lipids, amino acids, and a variety of other biomolecules. Consequently, central carbon metabolism has become a key metabolic process that viruses target and reprogram after infecting host cells. As obligate intracellular parasites, viruses require the hijacking of the host cell’s metabolic network to complete their own replication [7,8]. In order to secure a competitive edge for survival, viruses actively reprogram and co-opt the host’s metabolic pathways to optimize their replication. A prevalent mode of cellular subversion employed by viruses involves the alteration of the host cell’s glycolytic flux, culminating in the Warburg phenomenon. This is typified by an augmented rate of anaerobic glycolysis within the cellular milieu. The Warburg effect is not merely an enhancement of glycolytic activity; it is typically accompanied by an increase in glutaminolysis, the metabolic breakdown of glutamine [9]. In summary, viral molecules promote viral replication by inducing metabolic reprogramming in the host, and studying the connection between viral infection and host metabolism is a new direction for the development of antiviral drugs and diagnostic reagents. Targeting immune cell functions through metabolic modulation is a promising avenue for future immunotherapy [10].

Viral infections often accompany the reshaping of the host’s metabolism, with changes in carbon metabolism being the most common. However, the impact of FCV infection on host cell metabolism has not yet been studied. In order to further elucidate the role of host cell metabolism in the proliferation of FCV and to preliminarily explore the potential mechanisms by which FCV induces metabolic reprogramming in host cells, we employed unbiased metabolomic and transcriptomic sequencing to detect changes in central carbon metabolism-related metabolites and gene expression in FCV-infected CRFK cells. The results indicate that the infected cells increased their uptake of glucose and glutamine, with significant and concentrated changes in the abundance of central carbon metabolism and related products, indicating a reshaping of the central carbon metabolic pathway. The use of metabolic blocking drugs to block glycolysis, the pentose phosphate pathway, and glutamine metabolism effectively inhibited FCV replication in CRFK cells. Glutamine and glucose depletion experiments proved that both glutamine and glucose are essential for FCV proliferation. In summary, FCV infection reshapes the central carbon metabolic characteristics of CRFK cells to meet its own replication needs. Hence, targeting CCM may represent an innovative metabolic therapeutic target for FCV, facilitating the development of anti-FCV infection drugs.

## 2. Materials and Methods

### 2.1. Cell Culture and Viral Infection

CRFK cells were purchased from Wuhan Pricella Biotechnology (Wuhan, China). CRFK cells were cultured in DMEM (Wuhan, China) supplemented with 10% heat-inactivated fetal bovine serum (FBS) at 37 °C in a humidified 5% CO_2_ incubator. The FCV023 strain is isolated and preserved by our laboratory (GenBank: PP112969).

### 2.2. Reagents

DMEM was purchased from Wuhan Pricella Bio-technology(Wuhan, China). 2-Dexoy-D-Glucose (2-DG), Sodium Oxamate, 6-Aminonicotinamide (6-AN), CB-839, were obtained from Topscience (Shanghai, China) and diluted in accordance with the instruction. Monoclonal antibodies targeting the FCV VP1 protein were purchased from Absolute Antibody in the USA. Goat anti-rabbit IgG (H+L) Cross-Adsorbed Secondary Antibody, Alexa Fluor488, and Actin Muscle Monoclo-nal Antibody were purchased from Invitrogen (Carlsbad, CA, USA).

### 2.3. RNA Extraction and Real-Time Quantitative Polymerase Chain Reaction

Total RNA from cells was extracted with TRizol (St. Louis, MO, USA) and used as the template for reverse transcription. cDNA was synthesized using RevertAid First Strand cDNA Synthesis Kit (Waltham, MA, USA). q-PCR experiments were carried out using AceQ qPCR Probe Master Mix and AceQ qPCR SYBR Green Master Mix (Nanjing, China) according to the manufacturer’s instructions to measure transcripts or FCV RNA levels. The level of mRNA expression was normalized with β-actin. The sequences of primers are listed in (Table 1), and the qPCR reaction protocol is shown in Table 2.

### 2.4. Western Blotting Assay

Cells were lysed with RIPA Lysis and Extraction Buffer (Waltham, MA, USA) containing PMSF (Waltham, MA, USA) protease inhibitor. The titer of total protein was quantified using Protein Reagent Assay BCA Kit (Waltham, MA, USA). Western blot analysis was carried out according to the standard method. Antibodies used in this research were as follows: rabbit anti-FCV VP1 (Wilton Centre, UK) at a ratio of 1:2000; rabbit anti-β-actin (Waltham, MA, USA) at a ratio of 1:2000 [11].

### 2.5. The 50% Tissue Culture Infectious Dose (TCID_50_) Assay

The Reed-Muench method was used to determine the titer of FCV. CRFK cells were seeded into a 96-well plate. Subsequently, the FCV viral solution was serially diluted by a factor of 10 and used to infect the cells, with each dilution having 8 replicates. After a 72 h incubation period, the number of wells exhibiting cytopathic effects (CPEs) was recorded, and the viral titer was calculated [12].

### 2.6. Metabolite Extraction and LC-MS Analysis

Six biological replicates in FCV or mock-infected CRFK cells were set up for metabolomics detection. Extraction of metabolites and LC-MS analysis were carried out by Majorbio (Shanghai, China). The metabolites with variable importance in the projection (VIP) ≥ 1 and *p*-value of < 0.05 according to Student’s *t*-test were considered as significantly changed [13]. This part of the detection process was completed at Shanghai Megi Biopharmaceutical Technology Co., Ltd. (Shanghai, China).

### 2.7. Cell Viability Assay

CRFK cells were seeded into 96-well plates at a density of 1 × 10^4^/well and incubated with DMEM containing 10% FBS overnight. Cell culture medium was then replaced with 2% FBS DMEM with different concentrations of inhibitors and incubated for 8 h. Then, 10 μL of Cell Counting Kit-8 (Beijing, China) solution was added to each well and incubated for 4 h. The absorbance of each well was measured at 450 nm using a microplate reader (Revolution Optics, LLC, Burlington , VT, USA). All experiments were performed in quintuplicate.

### 2.8. Statistical Analysis

Except for special statistical analysis of transcriptome and metabolome described above, other statistical analyses were carried out using GraphPad Prism version 9 (GraphPad Software Inc., La Jolla, CA, USA). Statistical comparisons were carried out using Student’s *t*-test. *p*-values < 0.05 were considered significant (* *p* < 0.05,** *p* < 0.01, and *** *p* < 0.001).

## 3. Results

### 3.1. FCV023 Isolation Identification and One-Step Growth Curve Determination

**Figure 1:**
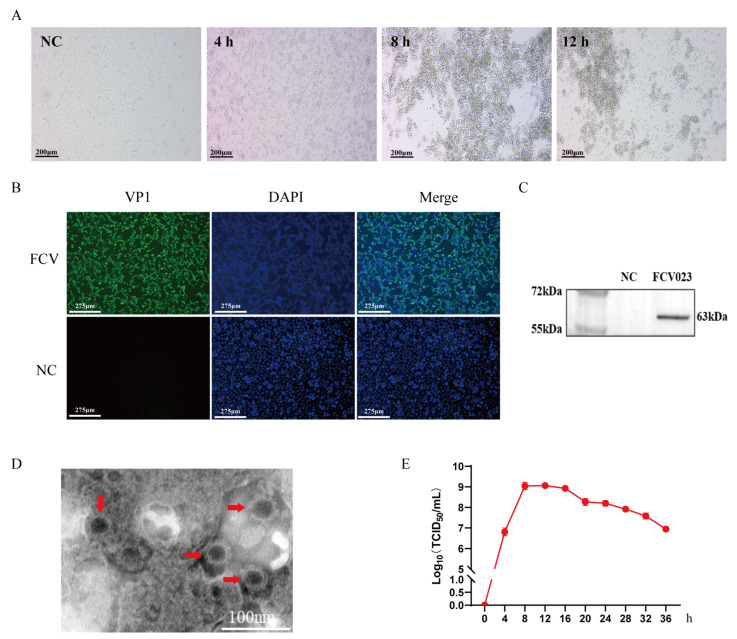
Identification of FCV in CRFK cells. (**A**) Cytopathic effect (CPE) of CRFK cells infected with FCV. (**B**) Immunofluorescence detection results 24 h after inoculation. Scale bar =  275 μm. (**C**) Western blot analysis of total cell lysates from virus-infected cells using anti-FCV VP1 polyclonal antibodies. (**D**) Electron micrograph of FCV virions (arrow heads) in cell culture media of infected CRFK cells. Scale bar = 100 nm. (**E**) One-step growth curve analysis of CRFK cells infected with FCV023 strains over 36 h; measurement at each time point was conducted in triplicate, and each experiment was set with three parallel groups. Average value was taken as TCID50 of virus. (Figure S1).

FCV023 was inoculated into CRFK cells, and cytopathic effects (CPEs) were observed at 4 h post-inoculation. The affected cells exhibited pyknosis and detachment (Figure 1A). Viral supernatants were harvested and subjected to a Western blot analysis and immunofluorescence assay (IFA) using FCV-specific monoclonal antibodies, which detected a protein band of approximately 62 kDa corresponding to the VP1 capsid protein and revealed FCV-specific fluorescent signals (Figure 1B,C). Transmission electron microscopy (TEM) was employed to visualize the viral particles. As depicted in Figure 1D, non-enveloped calicivirus-like particles with a characteristic cup-shaped morphology were observed, consistent with the structural features of caliciviruses, confirming the replication and proliferation of FCV023 in CRFK cells.

The titration of the FCV023 virus was performed using the Reed-Muench method. FCV023 was inoculated into CEFK cells at a multiplicity of infection (MOI) of 1, with culture supernatants collected at 4 h intervals for virus titer (TCID_50_) determination. The virus titer of FCV023 peaked at 8 h post-infection with CRFK cells, remained stable with no significant changes from 8 to 12 h post-infection, and then gradually declined after 16 h of cultivation (Figure 1E).

### 3.2. Metabolomic Studies Have Identified That FCV023 Infection Affects Central Carbon Metabolism in CRFK Cells

**Figure 2:**
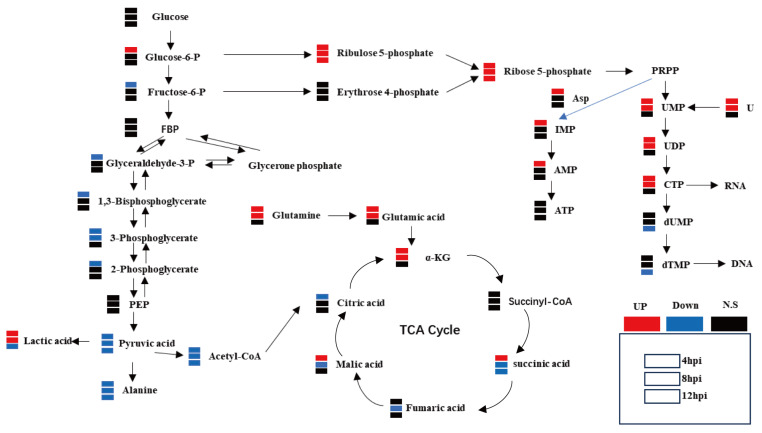
FCV023 infection remodels the central carbon metabolism in CRFK cells. The abundance changes in major intermediate metabolites in the central carbon metabolism of CRFK cells during FCV023 infection.

Metabolomic analyses were conducted on CRFK cells infected with FCV023 at 4, 8, and 12 h post-infection. The results reveal that during FCV infection, the central carbon metabolism in CRFK cells underwent significant reorganization, with notable alterations in the abundance of numerous metabolites within the central carbon metabolic pathways. In the early phase of FCV infection, compared to the mock-infected controls, the levels of glycolytic intermediates, such as pyruvate, 3-phosphoglycerate, and fructose-6-phosphate, were downregulated, while the abundance of the end product lactate was upregulated. The content of glucose-6-phosphate was significantly elevated, and there were marked increases in the abundance of metabolites such as ribulose-5-phosphate and ribose-5-phosphate, indicating an upregulation of the pentose phosphate pathway flux. Additionally, the levels of α-ketoglutarate in CRFK cells infected with FCV023 were significantly increased, coinciding with the enhanced metabolism of glutamate and glutamine, with a significant upregulation of related metabolite levels in infected cells. However, the intracellular ATP levels in infected cells remained unchanged compared to those in the uninfected cells (Figure 2).

### 3.3. 2-DG and Sodium Oxamate Reduces FCV023 Infection in CRFK Cell

Metabolic and transcriptomic analyses of CRFK cells infected with FCV023 indicate that during viral infection, anaerobic glycolysis and PPP flux are increased, while the acetyl-CoA levels are significantly decreased, and the intracellular ATP levels are slightly upregulated. This suggests that the total amount of acetyl-CoA generated from glucose metabolism entering the TCA cycle is downregulated, and other energy metabolic substrates maintain the cellular energy supply. To further determine the role of each central carbon metabolic segment in FCV infection and whether glycolysis favors the replication and proliferation of FCV, CRFK cells were treated with glycolysis inhibitors 2-DG and Sodium Oxamate and then infected with FCV023, followed by the detection of viral genome copies and the production of progeny virus.

**Figure 3:**
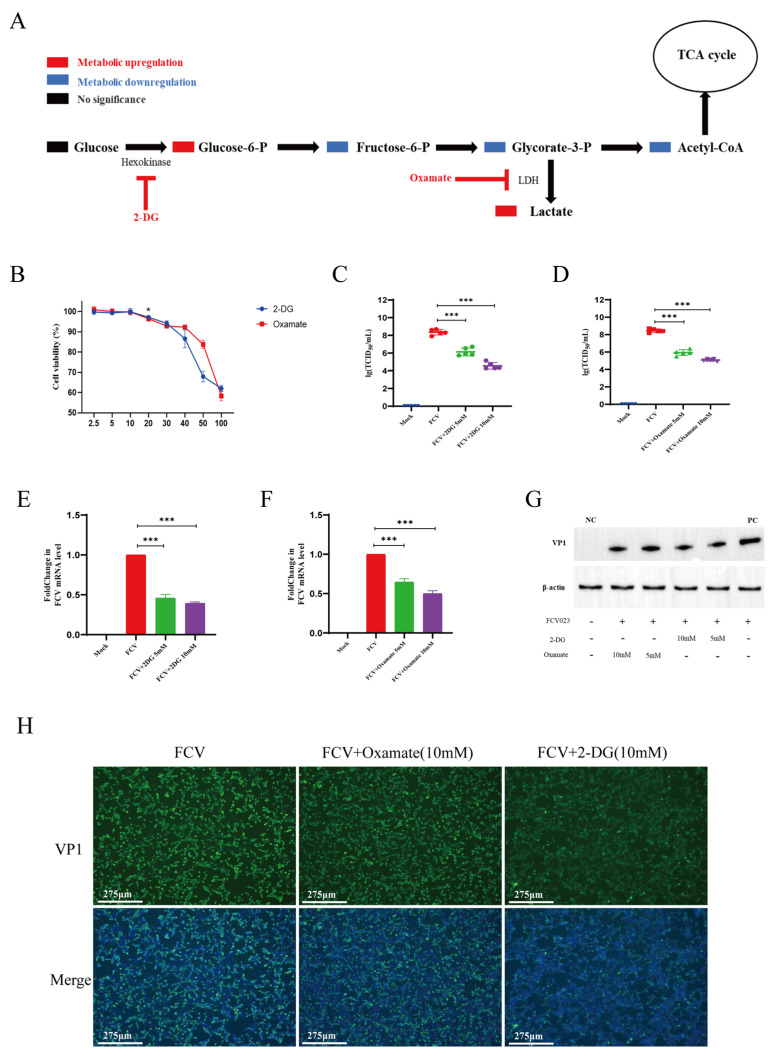
The inhibition of glycolysis inhibits the replication of FCV. (**A**) A schematic diagram of the functional targets of glycolytic pathway inhibitor drugs (2−DG, Sodium Oxamate) used in this study. (**B**) The impacts of different final concentrations of inhibitory drugs on the viability of CRFK cells after 8 h of treatment. (**C**,**D**) FCV023 infects CRFK cells at an MOI of 1 and is incubated in the presence or absence of glycolytic inhibitors, with viral titers being determined after 8 h. (**E**,**F**) FCV023 infects CRFK cells at an MOI of 1, and the intracellular mRNA levels of the FCV VP1 gene are analyzed by q−PCR after incubation in the presence or absence of glycolytic inhibitors for 8 h post-infection. (**G**,**H**) FCV023 infects CRFK cells at an MOI of 1 and is incubated in the presence or absence of glycolytic inhibitors, with the expression of FCV VP1 protein being analyzed by Western blotting after 8 h of infection. *p* < 0.05 was considered as statistically significant, * *p* < 0.05, *** *p* < 0.01. (Figure S2).

The treatment of CRFK cells with 10 mM of 2-DG or 10 mM of Sodium Oxamate for 8 h did not inhibit CRFK cell activity (Figure 3A), but after 24 h, it reduced the viability of CRFK cells by approximately 28% and 30% (Figure S1), respectively. Cells were infected with FCV023 at an MOI of 1 for 1 h. After infection, the culture medium containing 10 mM of 2DG or 10 mM of Sodium Oxamate was added to exclude the direct impact of the compounds on viral particles. After an 8 h incubation period, plaque assays observed a significant reduction in the number of infectious viral particles in cells treated with 2DG and Sodium Oxamate, a significant downregulation in viral genome copy numbers, and a significant inhibition of FCV023 capsid protein expression due to the blockade of the glycolytic pathway (Figure 3B–D).

### 3.4. FCV023 Infection Enhances the PPP in CRFK Cells, and the Unobstructed PP Facilitates FCV Infection

**Figure 4:**
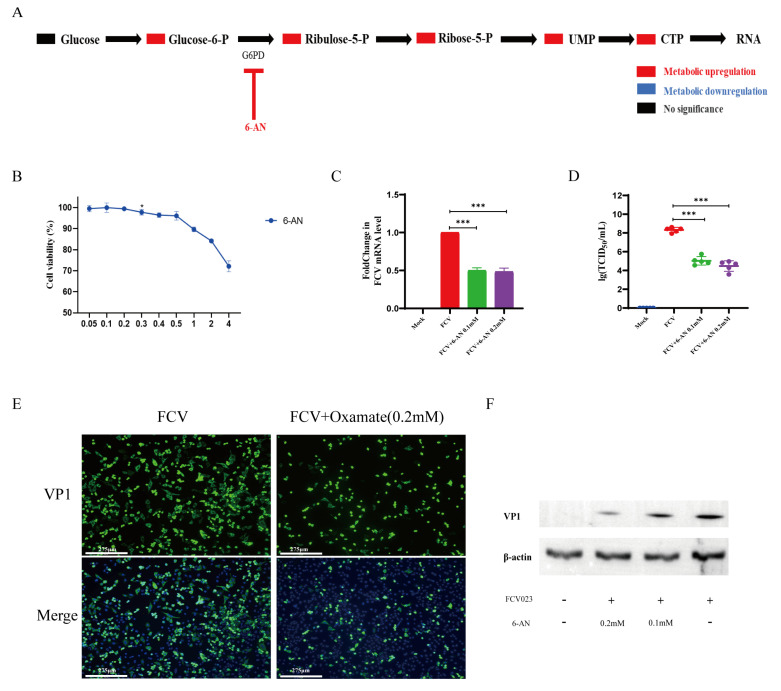
(**A**) A schematic diagram of the target sites of the PPP inhibitor 6-AN. (**B**) Changes in the viability of CRFK cells after treatment with 6-AN for 8 h. (**C**) The impact of 6-AN treatment on viral genome replication in FCV-infected CRFK cells (MOI = 1). (**D**) The impact of 6-AN treatment on viral titers in cells infected with FCV023 (MOI = 1). (**E**,**F**) FCV023 infects CRFK cells at an MOI of 1 and is incubated in the presence or absence of 6-AN, with the expression of FCV VP1 protein being analyzed by Western blotting after 8 h of infection. *p* < 0.05 was considered as statistically significant, * *p* < 0.05, *** *p* < 0.01. (Figure S3).

In general, PPP is the major branch pathway used by glycolysis for the de novo synthesis of nucleotides. Nucleotide metabolism plays a crucial role in viral replication, and we utilized a PPP inhibitor, 6-aminonicotinamide (6-AN), to ascertain the impact of the PPP on FCV virus infection. Cultured CRFK cells infected with FCV were treated with a final concentration of 1 μM of 6-AN, which demonstrated significant inhibitory effects on the viral genome level (Figure 4C), the release of infectious viral particles (Figure 4D), and the expression of capsid proteins (Figure 4E,F). This is likely due to the inhibitory effect of 6-AN on nucleotide synthesis, reducing the nucleotide precursors required for viral genome replication.

### 3.5. Glutamine Metabolism Is Crucial for the Proliferation of FCV023

**Figure 5:**
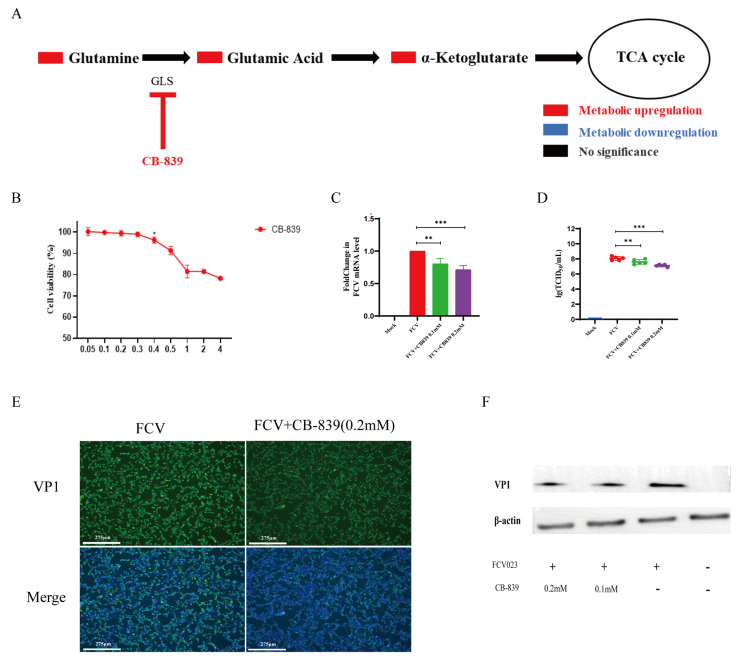
(**A**) A schematic diagram of the mechanism by which CB-839 inhibits glutamine metabolism. (**B**) Fluctuations in CRFK cell viability (incubation with CB-839 for 8 h). (**C**,**D**) The impact of treatment with 0.2 mM of CB-839 on viral genome replication and the release of infectious viral particles. (**E**,**F**) The effect of glutamine metabolism inhibition on the expression of FCV capsid proteins. *p* < 0.05 was considered as statistically significant, * *p* < 0.05, ** *p* < 0.01, *** *p* < 0.01. (Figure S4).

Glutamine metabolism is crucial for FCV023 infection as it serves as a secondary carbon source when glucose is scarce. It provides the necessary precursors for nucleotide synthesis, which are critical for viral genome replication. The metabolic intermediates of glutamine also support the TCA cycle, aiding in the maintenance of cellular energy supply. Furthermore, glutamine-derived carbon can be used for biosynthetic pathways, ensuring the normal metabolic functions of the cell. Subsequently, at 8 h post-infection, viral genome copies, capsid protein expression, and viral titers were assessed. In CRFK cells, the incubation of CRFK cells with a final concentration of 0.2 mM of CB-839 significantly inhibited the replication and copy of the FCV viral genome (Figure 5C), reducing the release of infectious viral particles (Figure 5D) and protein expression (Figure 5E,F).

## 4. Discussion

Viruses, as obligate intracellular parasites, rely on hijacking the host cell’s metabolic network to facilitate their replication. To establish a survival advantage, viruses actively modulate and exploit the host’s metabolic pathways to maximize their proliferation [14,15]. A common tactic employed by viruses is to stimulate glycolysis in host cells, leading to the Warburg effect, characterized by enhanced anaerobic glycolysis. The Warburg effect is not merely an amplification of glycolysis; it often involves intensified glutaminolysis. Central carbon metabolism, the means by which organisms derive energy, can rapidly supply energy and provide metabolic precursors for the synthesis of lipids, amino acids, and other biomolecules [16,17]. Consequently, central carbon metabolism becomes a focal point for hijacking and reprogramming by viruses following the infection of host cells. Viral infections lead to metabolic remodeling that promotes viral survival and replication, modulates the biological functions of host cells, and determines cell fate. Therefore, exploring the relationship between viral infections and host cell metabolism is a new hotspot in the study of viral infection mechanisms and the development of antiviral drugs [18,19,20].

Targeting host metabolic pathways has emerged as a novel strategy for developing antiviral drugs, elucidating the mechanisms of viral infection and pathogenesis, and guiding vaccine design [21,22,23]. Currently, the prevention of feline calicivirus (FCV) mainly relies on vaccination. However, the high variability of the virus limits the effectiveness of vaccine-induced immune protection, and there are no effective therapeutic drugs available in clinical settings. In this study, we found that central carbon metabolism underwent significant metabolic reprogramming in FCV-infected CRFK cells, which may be closely related to FCV’s immune evasion and proliferation. This suggests that the central carbon metabolism pathway can be a novel drug target for combating feline calicivirus. In this study, the optimal infection time for FCV in CRFK cells was determined to be 8 h post-infection (hpi). During the single-step growth curve determination of FCV023, it was observed that CRFK cells exhibited cytopathic effects 4 h post-infection and reached peak viral titers 8 h post-infection, and almost all cells detached and died by 12 h post-infection. This indicates that FCV023 strongly remodels the host’s metabolic activity early in infection to complete its own replication. Therefore, cells were chosen for metabolomic sequencing 8 h post-infection when cell viability was relatively high and viral replication was sufficient.

Utilizing metabolomic sequencing, we identified unique carbon metabolic changes in CRFK cells during FCV023 infection. Infected cells exhibited a significant reorganization of central carbon metabolism, with a substantial upregulation of lactate levels, indicating an intensification of anaerobic glycolysis in CRFK cells post FCV023 infection. Additionally, there was a slight increase in the overall intracellular energy levels, with a marked elevation of α-KG within the TCA cycle, and enhanced glutamine metabolism emerged as the primary substrate for energy metabolism. Metabolites and gene expression associated with the PPP also showed a significant increase in infected cells; these phenomena were also observed in RAW264.7 cells infected with murine norovirus [24,25]. In summary, FCV023 infection induces a unique carbon utilization pathway in CRFK cells, with the infection being closely linked to central carbon metabolic processes.

Hexokinase catalyzes the conversion of glucose to glucose-6-phosphate, serving as a pivotal substrate for various carbon metabolic pathways, thereby influencing multiple aspects of carbon metabolism. In normal cellular metabolism, glucose-6-phosphate is metabolized through glycolysis to produce pyruvate, which can then be converted into lactate or acetyl-CoA, entering the TCA cycle. 2-DG, a glucose analog, acts as a competitive inhibitor of hexokinase, effectively suppressing CCM. However, recent studies have employed 2-DG as an inhibitor of glycolysis, a usage that warrants further consideration [25,26]. Given its inhibitory target, 2-DG should be considered an inhibitor of CCM. To verify the role of glycolysis in FCV023 infection, Sodium Oxamate was also used to inhibit lactate production, further analyzing the impact of glycolytic pathway blockade on FCV023 proliferation. The addition of both inhibitors resulted in the reduced replication of FCV. Under conditions that do not affect cell viability, the inhibitory effect of 2-DG on FCV is more pronounced compared to Oxamate. This may be due to the fact that glucose-6-phosphate is the initial substrate for multiple metabolic pathways in central carbon metabolism. The addition of 2-DG inhibits the production of glucose-6-phosphate, thereby affecting several metabolic pathways, including glycolysis and the PPP, leading to a more potent inhibitory effect.

Recent studies on noroviruses in the Caliciviridae family have shown that the replication of mouse norovirus (MNV) is inseparable from glycolysis and glutamine metabolism, and blocking glycolysis and glutamine metabolism leads to a reduction in the synthesis of non-structural proteins and viral RNA in MNV [24,25]. Similarly, we found the same phenomenon in feline calicivirus (FCV), where the proliferation of FCV depends on unobstructed glycolysis. Recent research indicates that lactate produced by glycolysis can interact with mitochondrial antiviral protein (MAVS), thereby reducing the interaction between MAVS and RIG-I, inhibiting the production of IFN and thus interfering with the cell’s antiviral response [27,28,29,30]. In FCV-infected CRFK cells, the upregulation of lactate dehydrogenase activity gene expression and the increase in the lactate content suggest that the accumulation of lactate induced by FCV infection may also play an important role in immune evasion, which may be the reason for the limited proliferation of FCV023 due to the inhibition of glycolysis.

During the analysis of central carbon metabolism-related metabolites in CRFK cells infected with FCV, it was found that FCV infection induced a remodeling of central carbon metabolism. Numerous studies have indicated that after viral infection, glutamine metabolism is enhanced to maintain cellular energy supply, and a similar phenomenon was observed in FCV-infected cells. The blockade of both the pentose phosphate pathway (PPP) and glutamine metabolism significantly inhibits the proliferation of FCV. We believe that glucose is predominantly directed towards the PPP in FCV023-infected cells to generate nucleotides, which are then utilized for the synthesis of the viral genome. Meanwhile, glutamine serves as an alternative energy substrate, producing α-ketoglutarate (α-KG) to replenish the TCA cycle [31,32], thus maintaining cellular energy homeostasis. Glutamine and glucose hold a special metabolic position in FCV-infected CRFK cells, and their roles are yet to be further investigated.

This study confirms that FCV infection remodels cellular central carbon metabolism, with optimal FCV replication being dependent on unimpeded glycolysis, PPP, and glutamine metabolism. The inhibition of these metabolic pathways can significantly suppress FCV replication and the production of infectious viral particles. Therefore, the development of targeted drugs against these metabolic pathways may be a feasible direction for the development of specific drugs against FCV, which is of positive significance for the prevention and treatment of FCV. The experimental results also indicate that the metabolism of carbon and nitrogen sources in cells is closely related to FCV infection and pathogenesis; hence, a further assessment of the impact of glucose and glutamine deprivation experiments on FCV proliferation is necessary.

## 5. Conclusions

In conclusion, our study elucidates that FCV infection hijacks the central carbon metabolism of CRFK cells to ensure its replication. The infection alters the host cell’s metabolism of glucose and glutamine to enhance viral proliferation. The integrity of glycolysis, PPP, and glutamine metabolis m are key facilitators of FCV infection, and their inhibition significantly reduces FCV replication, demonstrating potent antiviral efficacy against FCV. This highlights the critical role of central carbon metabolism in FCV infection. Our research indicates that central carbon metabolism is a crucial link in FCV infection and represents a feasible therapeutic approach while also providing a new direction for the development of anti-FCV drugs.

## Figures and Tables

**Table 1:** The PCR primers used in this study.

Primer	Sequence (5′ to 3′)	Size
FCV-F	ATACCCGCCAATCAACATGT	84 bp
FCV-R	AGTCAATGTCAGGTGTCGGC	
Beta-actin-F	AAAGTCCAGGGCCACATAGC	400 bp
Beta-actin-R	AGGACTGCTATGTGGGGGAT	

Note: F is forward primer, R is reverse primer.

**Table 2:** The qPCR reaction protocol.

Stage 1	Pre-denaturation	Rep:1	95 °C	5 min
Stage 2	Cycling reaction	Rep:40	95 °C	10 s
			60 °C	30 s

## Data Availability

The nucleotide sequence of FCV023 (PP112969) has been uploaded to the GenBank, and the nucleotide information will be publicly available on 1 February 2025. The data that support the findings of this study are available upon request from the corresponding author.

## Supplementary Materials

The following supporting information can be downloaded at: https://www.mdpi.com/article/10.3390/vetsci12020138/s1, Figure S1: Original image of Figure 1C; Figure S2: Original image of Figure 3G.; Figure S3:Original image of Figure 4F.; Figure S4: Original image of Figure 5F.
