# Supplementary material for: Feline Calicivirus Infection Manipulates Central Carbon Metabolism
Source: Vet Sci. 2025 Feb 7;12(2):138. doi: 10.3390/vetsci12020138 (PMC11860418; doi:10.3390/vetsci12020138)
Supplement: Supplementary file 1 [file vetsci-12-00138-s001.zip › vetsci-3383714-supplementary.pdf]

Title: Feline calicivirus infection manipulates central carbon metabolism

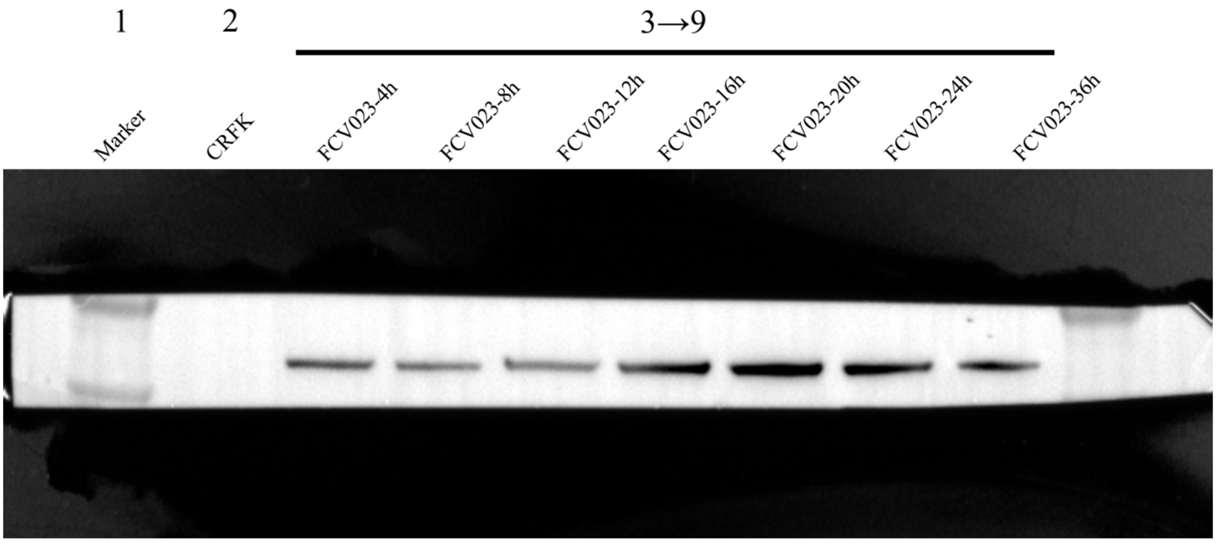

Figure S1. Original image of Figure 1C**.** Western Blot analysis was performed to examine cells infected with FCV023 at various time points post-infection. Lane 1 corresponds to the molecular weight Marker, Lane 2 represents non-infected cells, and Lanes 3 through 9 correspond to cells infected with FCV023.

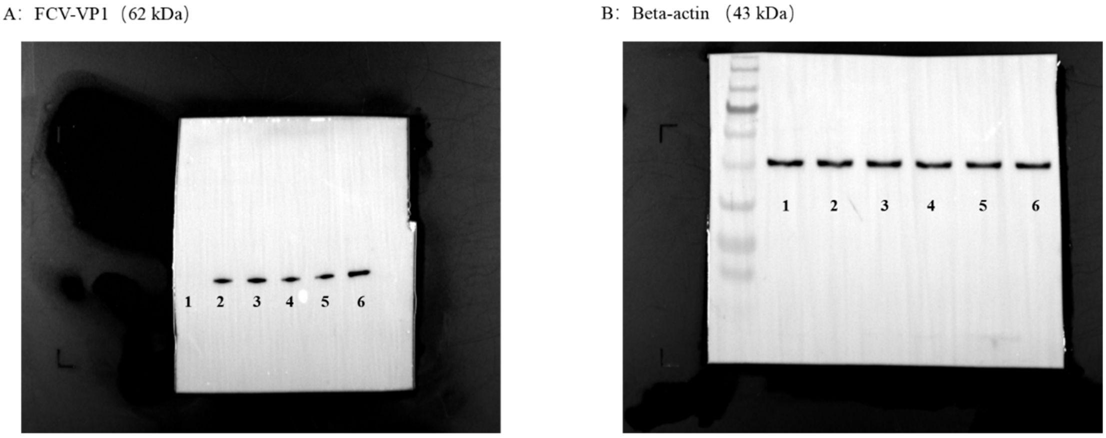

Figure S2. Original image of Figure 3G**.** Quantitative Western Blot analysis was utilized to detect the capsid protein of FCV023 in CRFK cells. Lane 1 represents uninfected CRFK cells. Lanes 2-6 represent CRFK cells infected with FCV023. Lanes 2 and 3 were treated with Oxamate at concentrations of 10mM and 5mM, respectively. Lanes 4 and 5 were treated with 2-Deoxy-D-glucose (2-DG) at concentrations of 10mM and 5mM, respectively, and Lane 6 was not subjected to any drug treatment.

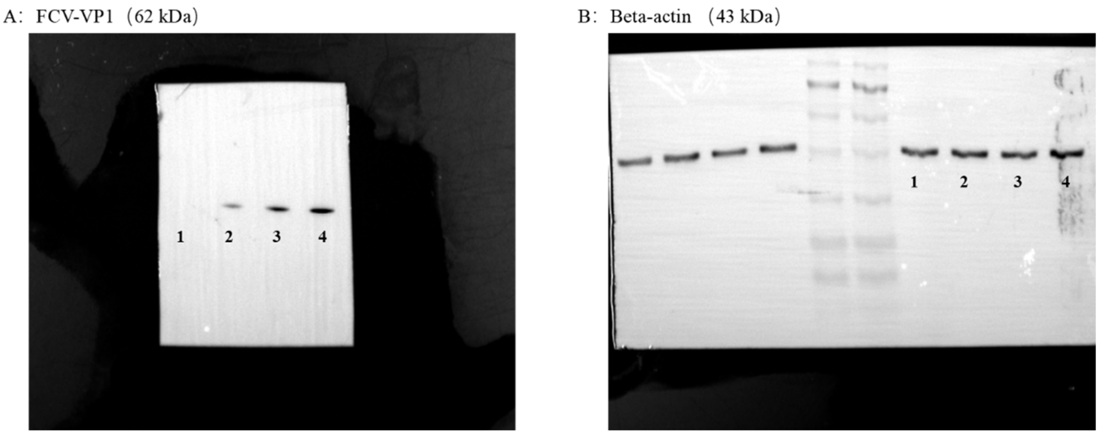

Figure S3. Original image of Figure 4F**.** Quantitative Western blotting was performed to assess the capsid protein expression levels of FCV023 in CRFK cells. Lane 1 contains non-infected CRFK cells. Lanes 2-4 contain FCV023-infected CRFK cells, with Lanes 2 and 3 treated with 6-Aminonicotinamide (6-AN) at concentrations of 0.2 mM and 0.1 mM, respectively. Lane 4 was not subjected to any treatment.

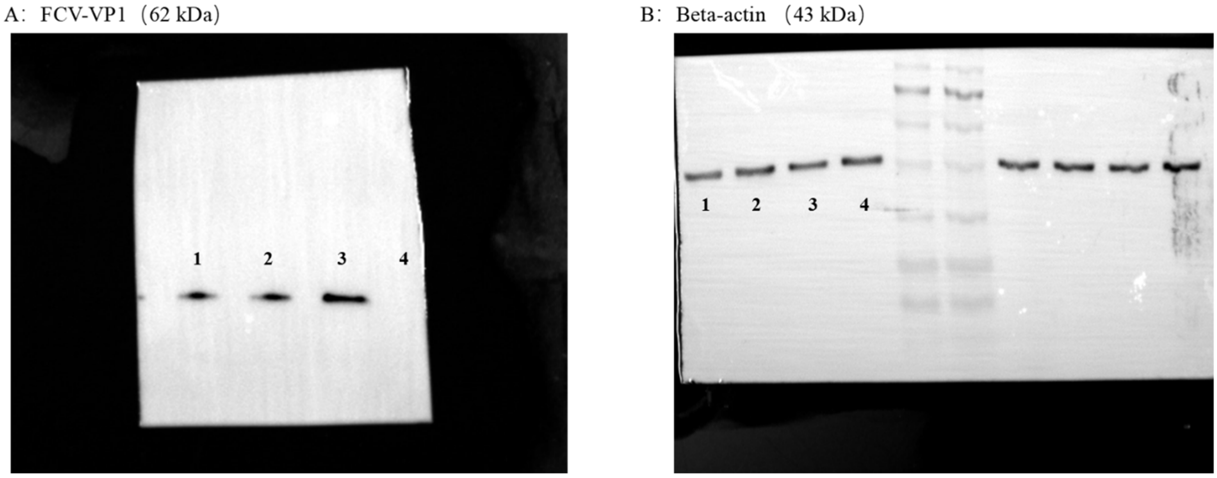

Figure S4. Original image of Figure 5F. Lane 4 contains non-infected CRFK cells. Lanes 1-3 contain FCV023-infected CRFK cells, with Lanes 1 and 2 treated with CB-839 at concentrations of 0.2 mM and 0.1 mM, respectively. Lane 3 was not subjected to any treatment.
